# Identification and optimization of PrsA in *Bacillus subtilis* for improved yield of amylase

**DOI:** 10.1186/s12934-019-1203-0

**Published:** 2019-09-17

**Authors:** Ane Quesada-Ganuza, Minia Antelo-Varela, Jeppe C. Mouritzen, Jürgen Bartel, Dörte Becher, Morten Gjermansen, Peter F. Hallin, Karen F. Appel, Mogens Kilstrup, Michael D. Rasmussen, Allan K. Nielsen

**Affiliations:** 1 Research and Technology, Novozymes A/S, Krogshoejvej 36, 2880 Basgvaerd, Denmark; 2 Institute for Microbiology, Department of Microbial Proteomics, Ernst-Moritz-Arndt-University Greifswald, F.- Hausdorff-Str. 8, 17489 Greifswald, Germany; 3 Technical University of Denmark, Søltofts Plads, Building 221, Room 204, 2800 Lyngby, Denmark

**Keywords:** *Bacillus subtilis*, Recombinant protein production, Secretion stress, PrsA

## Abstract

**Background:**

PrsA is an extracytoplasmic folding catalyst essential in *Bacillus subtilis*. Overexpression of the native PrsA from *B. subtilis* has repeatedly lead to increased amylase yields. Nevertheless, little is known about how the overexpression of heterologous PrsAs can affect amylase secretion.

**Results:**

In this study, the final yield of five extracellular alpha-amylases was increased by heterologous PrsA co-expression up to 2.5 fold. The effect of the overexpression of heterologous PrsAs on alpha-amylase secretion is specific to the co-expressed alpha-amylase. Co-expression of a heterologous PrsA can significantly reduce the secretion stress response. Engineering of the *B. licheniformis* PrsA lead to a further increase in amylase secretion and reduced secretion stress.

**Conclusions:**

In this work we show how heterologous PrsA overexpression can give a better result on heterologous amylase secretion than the native PrsA, and that PrsA homologs show a variety of specificity towards different alpha-amylases. We also demonstrate that on top of increasing amylase yield, a good PrsA–amylase pairing can lower the secretion stress response of *B. subtilis*. Finally, we present a new recombinant PrsA variant with increased performance in both supporting amylase secretion and lowering secretion stress.

## Introduction

Enzymes are used as catalysts to manufacture a variety of commercial products—like sugar, beer, bread, and ethanol. They are also used directly in products such as household care detergents, where they help remove stains and enable low-temperature and more sustainable laundry [1]. Many of these industrial relevant enzymes are produced in Gram-positive bacteria such as *Bacillus licheniformis* and *Bacillus subtilis* which are well known for high-level protein secretion and are generally regarded as safe [2, 3]. To achieve commercially relevant yields of enzymes, it is crucial to identify potential bottlenecks in the protein production route from transcription, translation to folding and secretion. Even though several heterologous proteins can be produced in these *Bacillus* species in very high yields, some enzymes cannot be secreted into the extracellular medium in titres high enough for their production to be economically cost-efficient. The bottlenecks that the production of heterologous proteins encounter can be found at different stages. In the cytoplasm, newly synthesized proteins can form insoluble aggregates and thus be degraded. On the membrane, they can be either poorly targeted or rejected by the preprotein translocation system. If the proteins are not folded quickly and correctly after translocation, they can be degraded by the several proteases that exist around the membrane, cell wall or extracellular medium. Two of the quality control proteases that degrade misfolded proteins around the cell-wall interface are HtrA and HtrB [4]. The expression of these is controlled by the CssRS two component system [5]. CssS is a sensor kinase that responds to overexpression of secretory proteins and heat stress by phosphorylating both itself and CssR, a cytoplasmic response regulator. The phosphorylated CssR activates the bicistronic CssRS operon and the *htrA* and *htrB* genes [6]. This response is referred to as secretion stress, which has been shown to be triggered by the secretion of heterologous alpha amylases, such as AmyQ from *Bacillus amyloliquefaciens*, or *B. subtilis*’ Lipase A [7].

Most exported bacterial proteins are translocated through the highly conserved SecA-YEG pathway [8]. Proteins are exported through this pathway in an unfolded state and to avoid proteolysis they must fold into their native conformation shortly after the signal peptide is cleaved, and leave the membrane. At this post-translocational stage, various thiol-disulphide oxidoreductases, negatively charged cell wall polymers and folding catalysts play a prominent role [9].

The major extra cytoplasmic folding factor in *Bacillus subtilis* is the PrsA protein [10]. PrsA belongs to the parvulin family of prolyl cis/trans isomerases (PPIases), which are ubiquitous in all types of cells and cell compartments, and catalyze rate-limiting protein folding steps at peptidyl bonds preceding proline residues [11]. PrsA and PrsA like proteins are widely conserved among gram-positive bacteria, and while it is essential for viability in many non-pathogenic *Bacillus* species, it is also involved in antibiotic resistance in gram-positives [12] such as *Staphylococcus aureus* [13], and it is essential for virulence and survival within the host cell in the pathogenic *Listeria monocytogenes* [14]. In addition, it has been shown that heterologous PrsAs are able to functionally complement complex activities such as flagellum-mediated swimming motility and pH resistance [15]. PrsA forms dimers in vivo and has two domains: PPIase domain and NC or chaperone domain [11]. The PPIase domain, which has a structure homologous to human Pin1, is the only one responsible for the PPIase activity of PrsA and it contains the hydrophobic proline-binding pocket [16]. The NC or chaperone domain is formed by the N-terminal (Ser4-Gly114) and C-terminal (Arg208-Ser260) regions and exhibits a motile clamp-like segment conserved among chaperones.

The PPiase domain is located near the NC domain, and both domains are joined by a short linker of 5 amino acids. This linker confers the PPIase domain rotational freedom from the chaperone domain. The dimeric form of PrsA is achieved solely by the interactions of the NC domains, and when dimeric, these form a highly hydrophobic bowl-like crevice surrounded by charged amino acids. This crevice, together with the two clamp regions, could sequester unfolded or unstable polypeptides of up to 20KDa, or even more considering the flexibility of both the NC and the PPI domains [11].

In *B. subtilis*, PrsA is a lipoprotein attached to the outer part of the membrane, and it is essential for cell viability due to its function on assisting the folding of the Penicillin Binding Proteins responsible for the synthesis of the cell wall [10]. PrsA may also take on other roles as there is now good evidence that it acts as a very efficient folding catalyst for exported amylases and various other enzymes [17–19].

In microbial cell factories, the yield of different amylases varies from one amylase to another even when industrial and biological settings are kept constant. PrsA foldases, when co-expressed with heterologous amylases, often enhances yield by supporting post-translocational folding and secretion of the product [19]. Expression of PrsA over wildtype level has in several studies proven beneficial for secretion of amylases such as AmyS (from *Geobacillus stearothermophilus*) [20], AmyL (from *Bacillus licheniformis*) [17] and AmyQ (from *Bacillus amyloliquefaciens*) [19]. Even though the native PrsA can support the production of various heterologous enzymes in *B. subtilis*, it may not be the optimal choice of PrsA for enhancing secretion of heterologous amylases from this bacterium. There are several examples that overexpression of the native PrsA may increase, decrease or not affect the secretion of the heterologous protein being produced, depending on the target exoprotein [20]. The *B. subtilis* amylase (*amyE*) evolved in the same host as its cognate PrsA and may, therefore, have a better fit to this PrsA than heterologous amylases.

Nature offers a wide range of both amylases and PrsA foldases and choosing the right match may increase the frequency of productive interactions between the enzyme and its foldase. For heterologous expression of amylases, it is most obvious to co-express the cognate *prsA* gene. The choice is more open in cases with engineered amylases, but a *prsA* gene from an organism close by in the evolutionary tree would be a good starting point.

In this study we set out to co-express six heterologous PrsAs with their cognate amylases, using *B. subtilis* as host. The heterologous amylase–PrsA pairs were equipped with their native signal peptides but were expressed from identical, non-native promoters at chromosomal locations. Furthermore, we constructed a matrix of strains containing all possible combinations of the six amylase-PrsA pairs and studied the importance of choice of PrsA for production of specific amylases. We show how the different heterologous PrsAs show diversity of specificity against the different amylases. In addition, we found that the cognate PrsA results in the highest increase in production in most cases. Moreover, we demonstrate how a good PrsA–amylase match is not only capable of increasing the amylase yield, but also relieves secretion stress. Lastly, we present a new recombinant PrsA that shows better performance on both improving AmyL secretion and on relieving secretion stress.

## Results

### The evolutionary relationship of amylase and PrsA variants

**Fig. 1:**
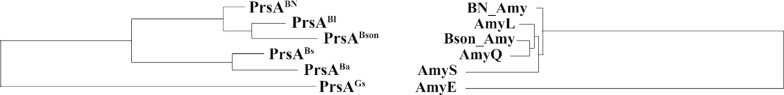
The interrelationship between members of the PrsA family proteins used in the study (left) and between the members of the AmyE family used (right). The phylogenetic tree was based on the mature protein sequence excluding signal peptides and calculated with the Phylogeny.fr tools (http://www.phylogeny.fr/) [24]. Branch lengths are proportional to the divergence of the amino acid sequences within each tree

The evolutionary relationships of the PrsA and alpha-amylase homologs used in this study are shown in Fig. 1. The sequence divergence within our set of alpha-amylase homologs is in general higher than what is found within the PrsA homologs which appear to be more conserved. Among bacterial proteins, essential genes appear to be more conserved than non-essential genes [21]. The *B. subtilis* AmyE protein is however atypical since it is quite distantly related to the AmyE homologs from the other closely related *Bacillus* species used in the study. This is also evident from the classification of GH13 amylases in the Carbohydrate Active Enzyme Database (CAZY) which has subdivided the GH13 family into subfamilies and mapped the *B. subtilis* AmyE to family GH13_28, and the rest of the AmyE homologs to the GH13_5 amylase family [22, 23]. The two amylase families are primarily distinguished by the presence of a large C-terminal carbohydrate binding domain in the GH13_28 members, which is completely absent in the members of the GH13_5 family. Of phylogenetic significance is also the presence of a large number (15 in the aligned area, shown in Additional file 1: Figure S1) of deletions spanning from 1 to 60 amino acids, in the amino acid sequence of the common domains of the GH13_5 family compared to the GH13_28.

Indels, defined as insertions or deletions are strong phylogenetic markers [25] and show that the division of the two amylase families did not happen late in the amylase evolution. Accordingly, a deep branching is found between AmyE and the GH13_5 family members (Fig. 1, right panel). Aside from *B. subtilis*, some isolates of *B. amyloliquefaciens* (not AmyQ), and *B. atrophaeus* contain amylase genes belonging to the GH13_28 family. The closest homologues to this atypical *Bacillus* amylase gene cluster are in *Streptococcus* isolates, while the genes in *Clostridium* isolates are somewhat more distant relatives. This atypical relationship is not observed for the PrsA proteins in the same organisms listed in Table 1. Here all PrsA proteins share a high degree of similarity, with only a few indels present (Additional file 1: Figure S2). Here *Geobacillus stearothermophilus* harbors the PrsA protein that diverts the most, as expected from the general phylogenetic relationship of the six bacterial species shown in Fig. 1 and Table 1.

**Fig. 2:**
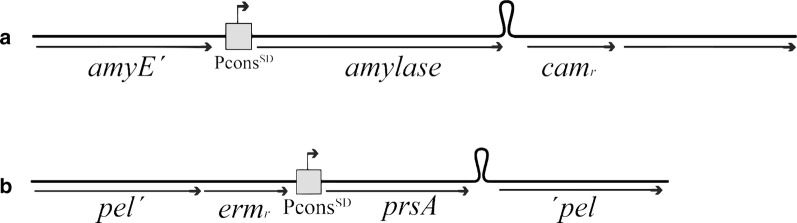
Expression cassettes for amylase and prsA expression. **a** The expression cassette for amylase expression containing the *amyE* locus 5′ and 3′ regions for homologous recombination, the PconsSD promoter followed by the amylase gene and the *amyQ* terminator. **b** Expression cassete for *prsA* expression containing the pel locus 5′ and 3′ regions for homologous recombination, the PconsSD promoter followed by the *prsA* gene and the native terminator of *B. subtilis prsA*

**Table 1:** PrsA (A) and amylase (B) identity matrices

	*B. subtilis*	*B. licheniformis*	*B. amyloliquefaciens*	*G. stearothermophilus*	*B. sonorensis L12*	*B. sp. NSP9.1*
(A) PrsA						
* B. subtilis*	100	66	85	55	67	70
* B. licheniformis*	66	100	66	50	85	86
* B. amyloliquefaciens*	85	66	100	54	66	69
* G. stearothermophilus*	55	50	54	100	51	55
* B. sonorensis L12*	67	85	66	51	100	84
* B. sp. NSP9.1*	70	86	69	55	84	100
(B) Amylase						
* B. subtilis*	100	35	25	31	35	30
* B. licheniformis*	35	100	65	81	84	84
* B. amyloliquefaciens*	25	65	100	66	67	67
* G. stearothermophilus*	31	81	66	100	83	81
* B. sonorensis L12*	35	84	67	83	100	85
* B. sp. NSP9.1*	30	84	67	81	85	100

Percent sequence identity between (A) PrsA homologs and (B) amylase homologs of different species calculated based on the pairwise Smith and Waterman alignment of the mature protein sequence

This analysis shows that the evolutionary relationship between secreted amylases and their native extracellular PrsA foldases is different for the AmyE/PrsA pair in *B. subtilis* than for the AmyL/PrsA^Bl^, AmyQ/PrsA^Ba^, and AmyS/PrsA^Gs^ pairs in *B. licheniformis*, *B. amyloliquefaciens*], and *G. stearothermophilus*, respectively. It also raises doubts whether the *B. subtilis* AmyE would be more evolutionary adapted to the *B. subtilis* PrsA than AmyL, AmyQ, or AmyS. Therefore, the effect of different PrsA homologues on heterologous amylase production was investigated.

### Chromosomal organization and expression of *amyE/prsA* homologous in *B. subtilis*

Expression cassettes were constructed and integrated into the chromosome of *B. subtilis* AN2 as described in Experimental Procedures. PrsA expression cassettes were integrated into the pel locus and consisted of the synthetic promoter PconsSD followed by a *prsA* gene and the *B. subtilis prsA* native terminator. Expression cassettes for amylases were integrated into the *amyE* locus and consisted of the synthetic promoter PconsSD followed by an amy gene and the *B. amyloliquefaciens amyQ* terminator. All the elements remained constant between strains except for the open reading frame of the gene of interest in the expression cassettes (Fig. 2).

**Fig. 3:**
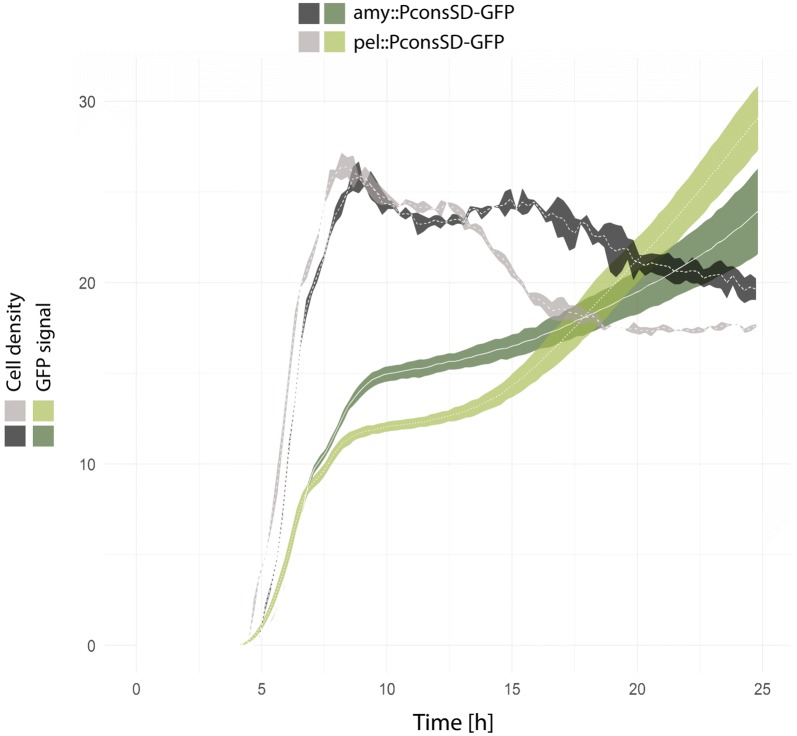
Gfp expression through growth. A *gfp* gene was inserted in the *amy* or *pel* locus in the same context as the amylase or *prsA* genes respectively to assess its expression profile. The GFP signal and the cell density were measured on-line in a Biolector plate reader

Initial experiments using strains which express gfp from the expression cassettes in either amy- or pel- loci verified the activity of PconsSD throughout the culturing period (Fig. 3). All strains used in this study also harbor the native *prsA* locus at its original location. Inactivation of the native *prsA* gene was only possible if preceded by the expression of either *B. subtilis*, $prsA^{{Bl}}$ or $prsA^{{Ba}}$. However, we observed no significant differences in amylase activities when these three strains were compared to their respective isogenic strains harboring the native *prsA* locus. This suggests that the activity arising from the native locus in these strains is overshadowed by the abundance of their homologous expressed from the strong PconsSD promoter in pel locus. A matrix of strains co-expressing all the combinations of the previously listed *prsA*’s and amylases, one to one, was constructed.

### Relative quantification of membrane PrsA protein levels by quantitative proteomics analysis

The strains expressing *amyL* from *B. licheniformis* from the amy locus and co-expressing each one of the previously listed *prsA*’s from the pel locus were selected for the measurement of the PrsA levels in the membrane fraction.

**Fig. 4:**
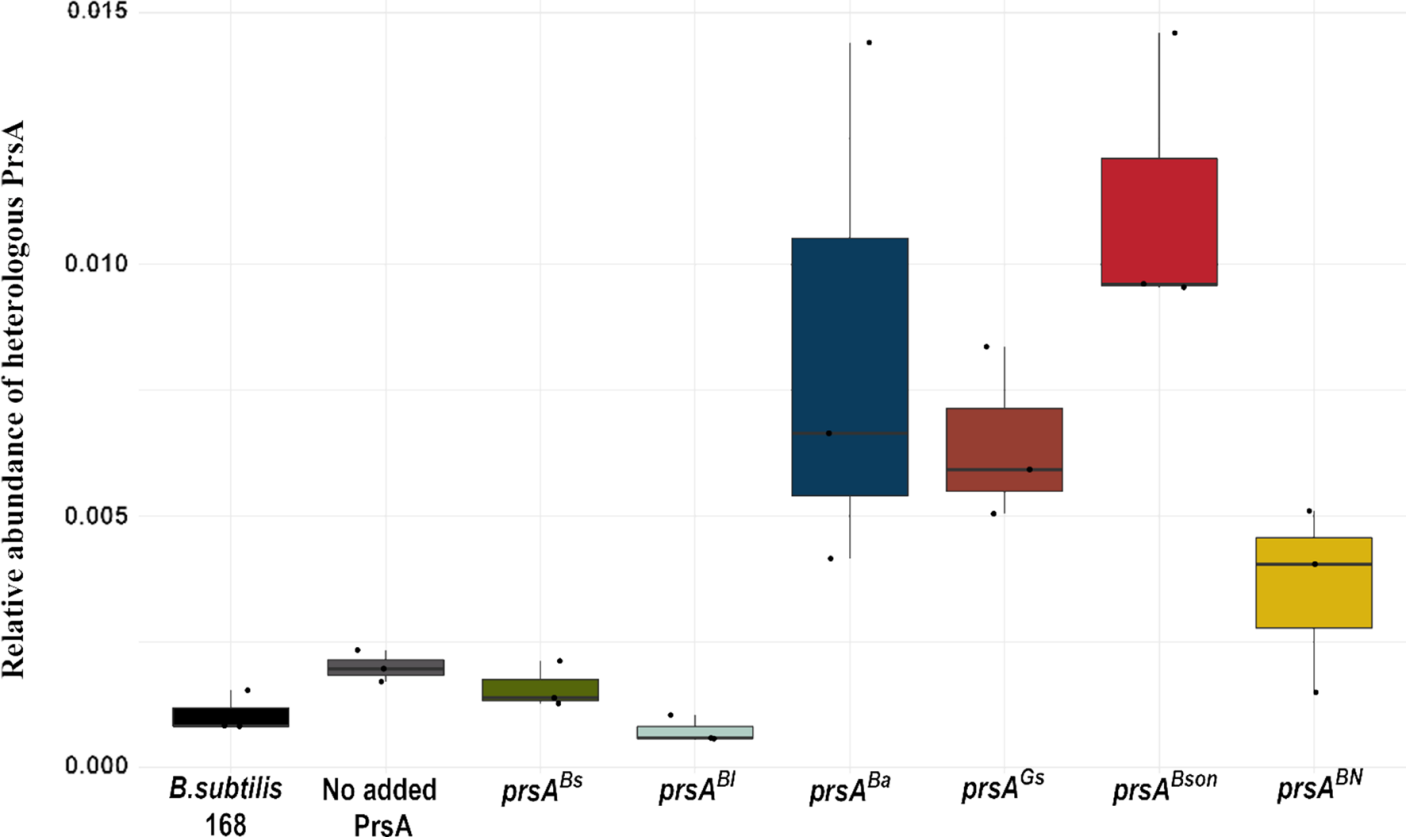
Relative abundance of heterologous PrsA in the cell membrane fraction. The membrane proteome of *B. subtilis* strains was analysed by LC-MS/MS and label-free Hi3 quantification. The relative amount of heterologous PrsA is given as the PrsA intensities divided by the sum of the total proteome intensities. All strains except *B. subtilis* 168 are expressing amyL. Levels of heterologous PrsA are shown except for B. subtilis 168 and No added PrsA strains, in which only the native PrsA is present. For the strain co-expressing a second copy of *B. subtilis* PrsA from the *pel* locus, the total amount of *B. subtilis* PrsA is shown

**Fig. 5:**
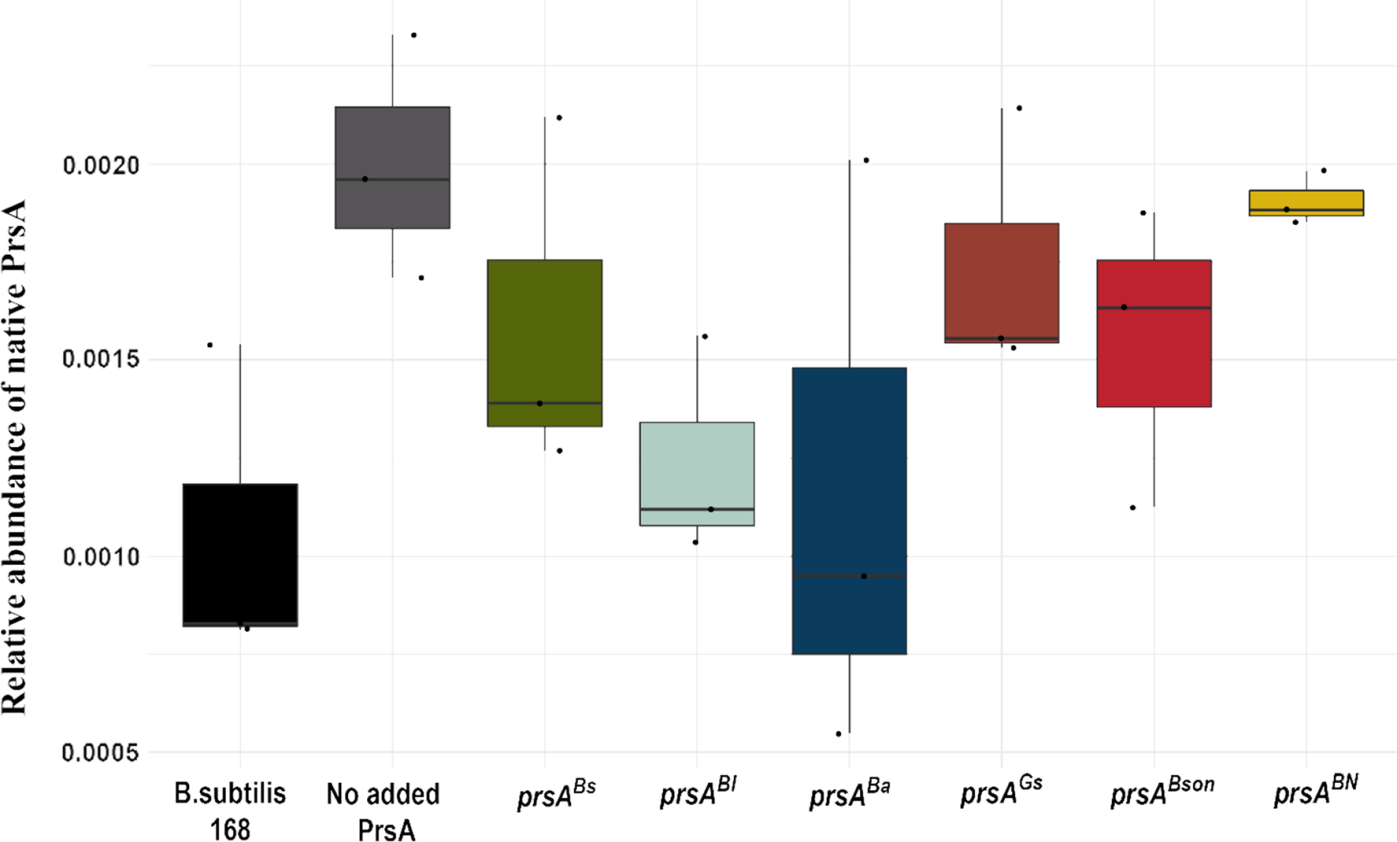
Relative abundance of native PrsA in the membrane fraction. The membrane proteome of *B. subtilis* strains was analysed by LC-MS/MS and label-free Hi3 quantification. The relative amount of native PrsA is given as the PrsA intensities divided by the sum of the total proteome intensities. All strains except *B. subtilis* 168 are expressing *amyL*

A quantitative proteomics analysis of the membrane fraction of all the AmyL expressing strains and the parental *B. subtilis* 168 $\Delta$*sigF* strain was performed by label-free quantification by LC-MS/MS. The Hi3 relative quantification method determines protein abundancies by adding up the signal intensity of the three most abundant peptides of each protein. The relative amount of PrsA is calculated by computing the Hi3 data of all strains and calculating the ratio of the PrsA intensities divided by the sum of the whole detected proteome intensities. This way the amount of the heterologous PrsAs relative to the total amount (or intensity) of all the proteins detected, can be compared with each other. In Fig. 4 the relative amounts of the heterologous PrsAs are shown, whereas the relative amounts of the native *B. subtilis* PrsA in each strain are shown in Fig. 5.

As can be seen in Fig. 4, all heterologous PrsAs are detected in the membrane. Nevertheless, the relative amount of each of them is not the same in all strains. This could be due to variations in either mRNA degradation, degradation in the cytoplasm, incorrect destination sorting or degradation after translocation through the Sec pathway. Regarding the native *B. subtilis* PrsA (Fig. 5), it is an interesting observation that the strain that overexpress AmyL but no heterologous PrsA has a twofold increase of native PrsA in comparison to the wildtype *B. subtilis 168* strain. The amount of the native PrsA is not increased in the strain overexpressing the *B. subtilis* PrsA, and it is surprisingly reduced in the strain co-expressing the cognate PrsA^Bl^ with AmyL. The same twofold increase in the amount of native PrsA can be seen in the strain co-expressing PrsA^BN^ with AmyL.

### Heterologous co-expression of *prsA* and amylase cognates and effect on amylase activity

Table 2 shows amylase activities measured in supernatants from each series of strains co-expressing a specific amylase and the various heterologous *prsA* genes included in this study. Values in each series are set relative to the strain that expresses the amylase from the *amyE* locus but which do not co-express any *prsA* from the pel locus. The table reveals that the highest amylase activities in most cases were obtained when the heterologous amylase was co-expressed with its cognate *prsA* gene in *B. subtilis*. Some non-cognate combinations of amylase and *prsA* genes were also observed to increase amylase activity other than the cognate ones, but none of these were superior to a cognate pair (Table 2). The only exception to this general observation was when the *G. stearothermophilus* amylase (AmyS) and PrsA were co-expressed in *B. subtilis*. In this case the extracellular amylase activity was lower than when the amylase was co-expressed with a *prsA* gene from either of *B. subtilis*, *B. licheniformis* ($prsA^{{Bl}}$), or *B. amyloliquefaciens* ($prsA^{{Ba}}$). Only in the case where AmyQ was co-expressed with the *G. stearothermophilus* ($prsA^{{Gs}}$) PrsA did we observe a severe negative effect of extra PrsA on amylase activity.

**Table 2:** Relative extracellular amylase activity in growth medium of *Bacillus subtilis* 168 $\Delta$*sigF* co-expressing heterologous amylases and heterologous *prsA* genes

Origin of heterologous prsA gene inserted into the pel locus							
Origin of heterologous amyE homologue inserted into the amyE locus	None (only wild type prsA gene)	+ *B. subtilis* prsA	+ *B. licheniformis* $prsA^{\mathrm{Bl}}$	+ *B. amyloliquefaciens* $prsA^{Ba}$	+ *G. stearothermophilus* $prsA^{Gs}$	+ *B. sonorensis* L12 $prsA^{Bson}$	+ B. NSP9.1 $prsA^{BN}$
*Bacillus subtilis (amyE)*	1 (0.09)	*1.25 ( 0.11)*	0.91 (0.06)	1.18 (0.13)	1.17 (0.18)	0.84 (0.07)	* 0.56 (0.16)*
*Bacillus licheniformis (amyL)*	1 (0.08)	1.20 (0.15)	*1.40 (0.08)*	*1.48 (0.06)*	*1.44 (0.08)*	*1.46 (0.10)*	1.06 (0.17)
*Bacillus amyloliquefaciens (amyQ)*	1 (0.08)	1.18 (0.13)*	*1.27 (0.18)*	1.19 (0.05)*	*0.29 (0.01)*	0.94 (0.007)	0.89 (0.02)
*Geobacillus stearothermophilus (amyS)*	1 (0.13)	*1.40 (0.22)*	*2.19 (0.15)*	*2.41 (0.21)*	1.01 (0.05)	0.93 (0.03)	*1.74 (0.03)*
*Bacillus sonorensis L12*	1 (0.1)	0.95 (0.28)	* 2.35 (0.41)*	*2.07 (0.25)*	* 1.72 (0.18)*	* 2.54 (0.19)*	* 1.94 (0.44)*
*Bacillus NSP9.1*	1 (0.17)	1.12 (0.26)	*1.77 (0.15)*	*1.64 (0.16)*	1.38 (0.1)	1.26 (0.13)	*1.50 (0.10)*

Values are calculated as the mean of six determinations, normalized to the level of amylase activity in the strain expressing each amylase with no added *prsA* gene. Results highlighted in italics are statistically significant ($p<0.05$) for a pairwise t-test Bonferroni corrected regarding the strain overexpressing the amylase with no added prsA expression. Results marked with an asterisk have a p value $0.10<p>0.05$

The development of biomass in cultures was monitored on-line and all grew alike except those expressing $prsA^{{BN}}$. These cultures ended up with optical densities approximately 40 percent lower than the cultures expressing other PrsAs likely due to increased cell lysis in their stationary phases (Additional file 1: Figure S3). The level of amylase activity in the supernatant was still comparable to other cultures indicating that PrsA^BN^ may be particularly good at supporting amylase secretion as compared to the other expressed homologs.

It is interesting to notice that the amount of PrsA found in the membrane and the effect of that PrsA on AmyL secretion does not seem to be directly connected. PrsA^Bl^, PrsA^Ba^, PrsA^Gs^ and PrsA^Bson^, despite appearing in different amounts in the membrane (Fig. 4), have the same positive effect on AmyL secretion (Table 2).

### Effect of PrsA in the secretion stress response

**Fig. 6:**
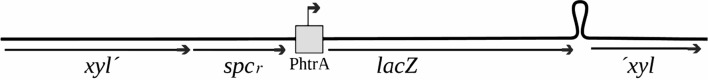
Expression cassette for measuring *htrA* induction. The expression cassette contains the *xyl* locus 5′ and 3′ regions for homologous recombination, the *PhtrA* promoter followed by the *lacZ* gene

Overproduction of amylases in *B. subtilis* has previously been reported to cause secretion stress. The cell responds by increasing the production of the quality control proteases HtrA and HtrB of the CssRS regulon which then remove misfolded proteins that would otherwise block the essential secretion machinery [5, 26]. To measure how co-expression of the various heterologous *prsA* genes with AmyL affects activity of the CssRS regulon, we employed a promoter fusion between the secretion stress inducible *htrA* promoter (P*htrA*) and the *lacZ* gene. The *PhtrA-lacZ* cassette was integrated into the xylose locus of the chromosome and the level of beta galactosidase activity was measured as an indicator for the degree of secretion stress (see Fig. 6 and experimental procedures).

**Fig. 7:**
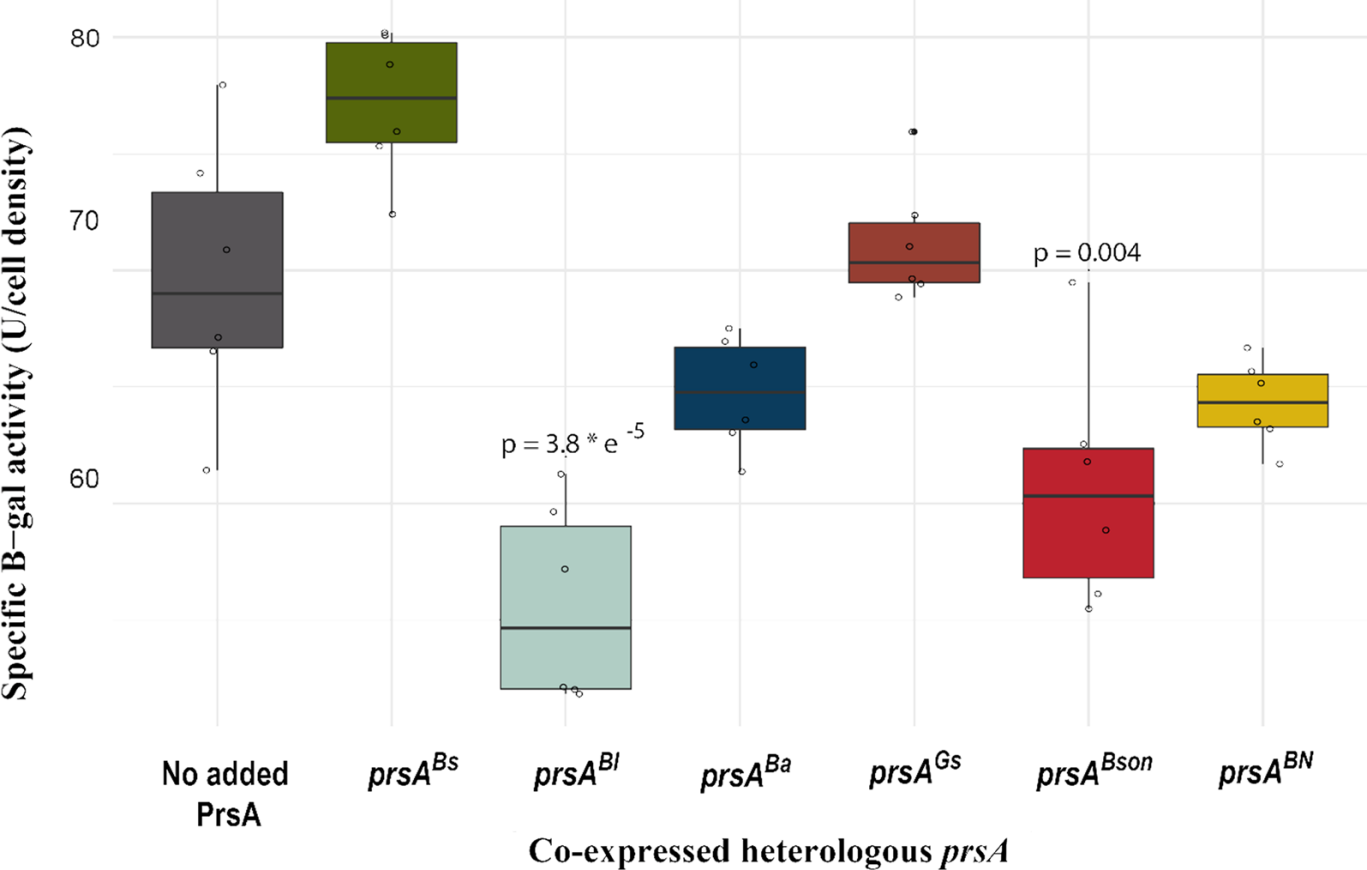
Specific $\beta$-galatosidase activity after 24 h. Two independent experiments with biological triplicates in each one were conducted and $\beta$-galatosidase activity was measured in the culture pellets. P-values are calculated by a pairwise T-test Bonferroni corrected

Figure 7 shows the beta-galactosidase activities measured in 24-h old cultures of the various *prsA* expressing strains, all expressing AmyL from the P*consSD-amyL* fusion used above. The figure reveals that co-expression of $prsA^{{Bl}}$ or $prsA^{{Bson}}$ with AmyL not only result in increased amylase activities in the supernatants (Table 2) but also leads to a significant decrease in activity of the *htrA* promoter. This observation may indicate that PrsA^Bl^ (the cognate PrsA) and PrsA^Bson^ both are able to support proper heterologous secretion of AmyL in *B. subtilis* and by doing so also decreases cellular secretion stress. The remaining four PrsAs, even those influencing AmyL secretion, do not seem to have any significant effect on the P*htrA* activity when co-expressed with AmyL. Figure 7 also reveals the interesting observation that a strain co-expressing amyL and $prsA^{{BN}}$ does not appear more secretion stressed than the reference strain with no extra *prsA*, suggesting that the increased cell lysis during the stationary phase is not a direct consequence of secretion stress.

### Engineering of PrsAs chaperone domain for improved amylase secretion

As noted previously we observed increased cell lysis in stationary phase cultures of all strains expressing $prsA^{{BN}}$ and this increased cell lysis was not correlated with a significant decrease in final total amylase activity. One potential and very interesting explanation to this could be the presence of specific structural or biochemical features in PrsA^BN^ that somehow facilitates superior amylase secretion, despite having a detrimental effect on cell growth. This observation led to the following question: would it be possible to modify PrsA^Bl^ to facilitate a superior specific amylase secretion, like the one observed with PrsA^BN^, while at the same time maintaining normal growth without cell lysis?

**Fig. 8:**
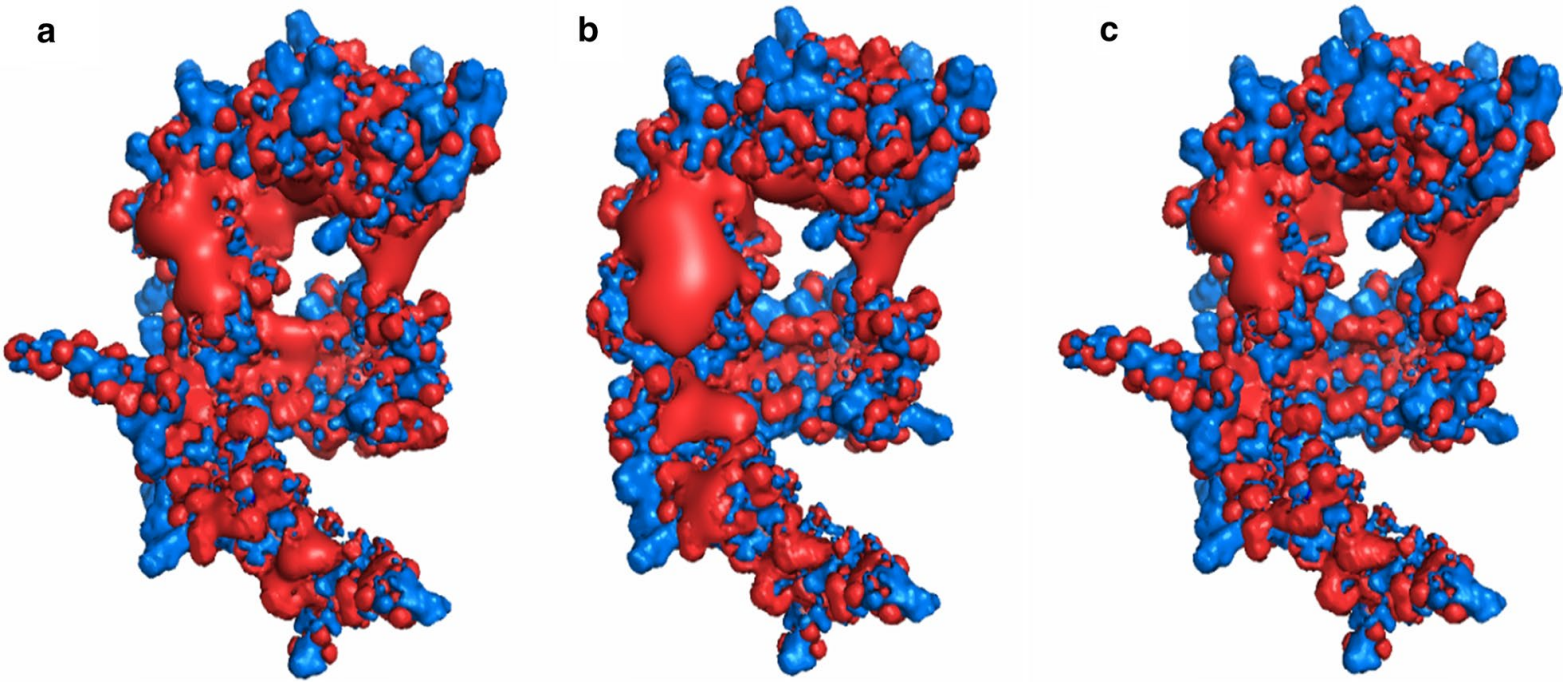
Modelled structures of PrsA$^\mathbf{Bl }$ and PrsA$^\mathbf{BN }$. The models were constructed by alignment to the known structure of *B. subtilis* PrsA (MUSCLE) and the calculation of the distribution of charges was done by PDB3PQR [27] and PROPKA for pKa calculations [28]. Positive charges are shown in blue and negative charges in red. **a** PrsA from *B. licheniformis*. **b** PrsA from *B. NSP9.1*. **c** Recombinant PrsA^Bl-BN^

**Fig. 9:**
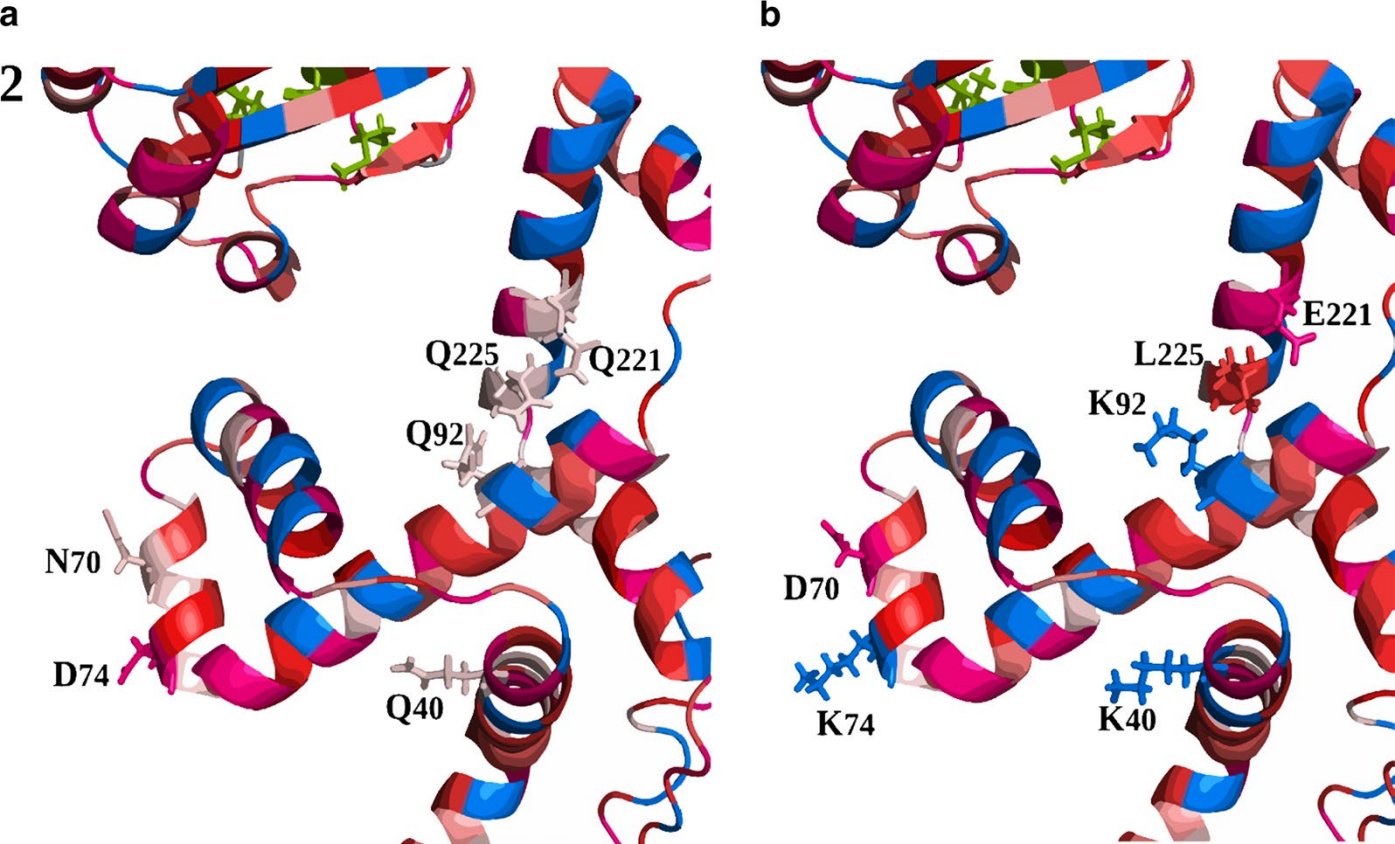
The six residues selected for substitution. The figure shows the NC domain of **a** PrsA^Bl^ and **b** PrsA^BN^. Positive charges are shown in blue, negative charges in pink. Red shows hydrophobicity

PrsA has two domains: the PPIase domain, responsible for the peptidyl prolyl cis-trans isomerase activity, and the NC or chaperone domain [11]. While the PPIase domains of the PrsA homologues are highly conserved, the NC domains are more variable [15] (Additional file 1: Figure S2). The latter differs greatly between species, both in sequence and molecular surface, which varies from very hydrophobic, as in *B. subtilis*, to very polar, as in *L. monocytogenes*’ PrsA1 [14]. This diversity on sequence and charge may reflect the diverse substrate specificities [11] and hence also explain the variations in yield of amylase we obtain dependent on choice of PrsA. It has been speculated that the bowl-like crevice formed by the NC domains of dimeric PrsA is involved in sequestration of unfolded or unstable polypeptides [11]. Dynamic interactions between the chaperone and its substrate would be highly dependent upon the charge distribution/electronegativity in the surface landscape in this region, and could influence the frequency of productive substrate interactions [14]. PrsA^Bl^ and PrsA^BN^ are very similar, differing in only 34 out of 286 positions (Additional file 1: Figure S2). Nevertheless, we observed a substantial difference in electronegativity when the NC domains of the two proteins were compared (Fig. 8). To bring the NC chaperone region of PrsA^Bl^ closer in structure to PrsA^BN^, six amino acids were substituted (Fig. 9). This new recombinant PrsA was named PrsA^Bl-BN^.

**Fig. 10:**
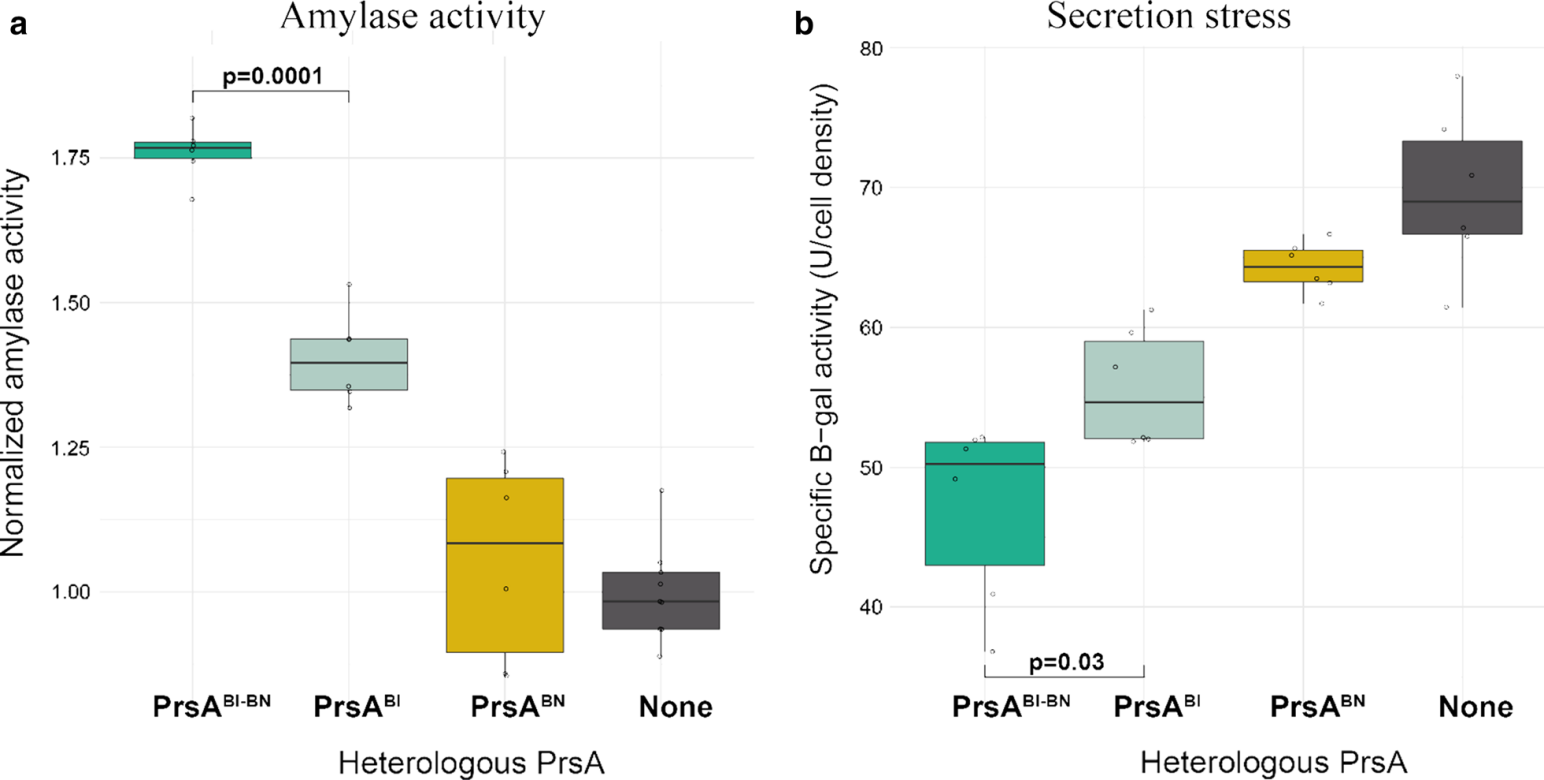
Total amylase activity and $\beta$-galactosidase activity after 24h of growth. Two independent experiments with three biological replicates were conducted in each case. P-values were calculated with a pairwise t-test Bonferroni corrected. **a** Total amylase activity in the supernatant. Results are normalized to the level of amylase activity in the strain expressing each amylase with no added *prsA* gene. **b** Specific $\beta$-galactosidase activity in the cell cultures after treatment with lysozyme. P-values are calculated by a pairwise T-test Bonferroni corrected

The recombinant prsABl-BN mutant was co-expressed with amyL and the yield of AmyL was compared to strains co-expressing amyL together with the $prsA^{{Bl}}$, $prsA^{{BN}}$, or just the wildtype levels of $prsA^{{Bs}}$ gene (Fig. 10a). The surface re-modeled PrsA^Bl-BN^ foldase increased total amylase activity compared to that of the native PrsA^Bl^ and PrsA^BN^ foldases. Furthermore, the strain expressing AmyL with this new recombinant PrsA^Bl-BN^ foldase appeared less secretion stressed than when AmyL was expressed with the native foldases, with an almost inverse correlation between AmyL yield and Secretion Stress level (Fig. 10). In addition, the strain expressing PrsA^Bl-BN^ showed no increased lysis in the stationary phase compared to the wildtype strain (Additional file 1: Figure S3).

**Fig. 11:**
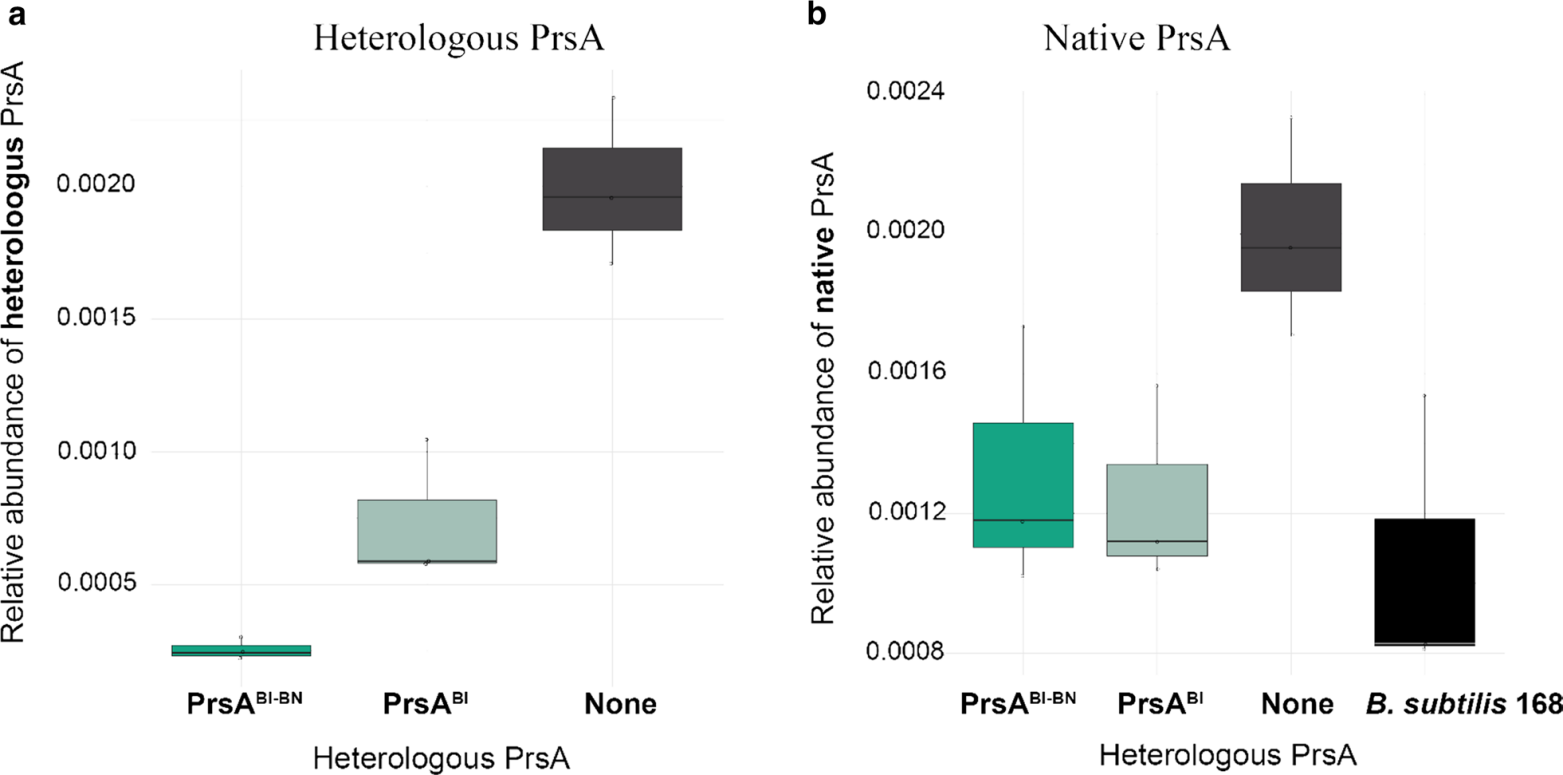
Relative abundance of PrsA in the membrane fraction. The membrane proteome of *B. subtilis* strains was analysed by LC-MS/MS and label-free Hi3 quantification. The relative amount of native PrsA is given as the PrsA intensities divided by the sum of the total proteome intensities. **a** Measured relative amounts of heterologous PrsA. **b** Measured relative amounts of native PrsA

Regarding the amount of PrsA present in the membrane, there is less PrsA^Bl-BN^ present than PrsA^Bl^, (Fig. 11a). There is also less heterologous PrsA in the membrane than native PrsA in both cases (Fig. 11a). As can be seen in Fig. 11b, in both cases the amount of native PrsA that can be found in the membrane is lower than in the strain expressing AmyL and no extra PrsA and there is no significant difference between the native amounts of PrsA between the two heterologous PrsA expressing strains.

## Discussion

In this study we set out to co-express six heterologous PrsAs with each of six heterologous amylases, using *B. subtilis* as a host. We constructed a matrix of strains containing all combinations of the six amylase–PrsA pairs and studied the importance of choice of PrsA for yield of amylases. Essential genes tend to be more conserved among bacteria than non-essential genes [21]. Even though correct folding of amylases is dependent on PrsA, the essential function of this foldase is however not related to the folding of amylases. The penicillin binding protein 2B (PBP2B), which in *B. subtilis* is an essential protein required for the synthesis of the cell wall, is dependent on PrsA for proper folding [10]. Thus, it may have been essential for PBP2B to evolve with a the cognate PrsA but there is no strict dependency of the amylase to evolve with the cognate PrsA.

Inactivation of the native *prsA* gene was only possible in strains expressing either *B. subtilis*, *B. licheniformis* or *B. amyloliquefaciens prsA*. This suggests that although a heterologous PrsA can complement *B. subtilis*’ native one, not all PrsAs can do so.* B. anthracis* PrsA has previously been shown to complement *B. subtilis* PrsA for both cell viability and heterologous protein secretion [29]. In *L. monocytogenes*, several different heterologous PrsAs can complement some of the native PrsA2 functions, like swimming motility, pH tolerance, and secretion of virulence factors, but not for others, like osmotic stress and cell wall biosynthesis [15]. So, even if some PrsA functions are very conserved among Gram positives, this foldase also appears to be very diverse in its substrate specificity. This may explain why some PrsAs can complement *B. subtilis*’ own, while others cannot, possibly because they don’t facilitate proper folding of the essential PBPs.

We expressed heterologous *prsA* genes in *B. subtilis* and measured their abundancies by relative quantification of the membrane proteomes. Different relative abundancies of the heterologous PrsAs were found in the membrane despite the fact that the different PrsAs were expressed from an identical genetic context. This could be due to several reasons: different levels of transcription, mRNA degradation, PrsA degradation in the cytoplasm, incorrect destination sorting, or degradation after translocation through the Sec pathway. Despite these differences, several PrsAs had identical effects on the yield of AmyL, suggesting that either the amount of PrsA is not relevant at this level or the different foldases fold the amylase with different efficiency.

If the reason for the differences in PrsA levels is incorrect sorting, some PrsA could accumulate in the cytosolic or extracellular fractions. Nevertheless, it has been shown that this foldase is only active in its dimeric form, and dimerization is only thought to occur when anchored to the membrane or present in the medium in very high concentrations ($750\,\mu \hbox {M}$) [11]. Therefore, it is unlikely that soluble PrsA present in the media could affect the folding of the amylases.

To our knowledge, there are no reports on the regulation of the *prsA* gene expression in *B. subtilis*. However, an interesting observation of this work was that the level of native PrsA was affected in some of the strains expressing amyL. The native PrsA level was twofold higher in the strain expressing amyL compared to the wild type *B. subtilis* 168 strain. We also noticed that the native PrsA level went back to normal when some heterologous PrsAs were co-expressed, but not others. Remarkably, the level of the native PrsA was back to normal when the cognate PrsA from *B. licheniformis* was expressed. We also observed that this cognate PrsA is superior to the other tested heterologous PrsAs when it comes to relieving secretion stress in AmyL producing strains. When looking at the genetic context in which the native PrsA gene is located, we identified two putative $\sigma$A promoters centered 60 (P1 promoter) and 92 bp (P2 promoter) upstream of the reading frame. Both promoters match 5 of the 6 canonical bases in their −10 and −35 boxes, and two transcriptional start sites (TSS’s) were mapped at exactly +1 in accordance with these locations. Superimposed on the −35 region of P1, we see a putative CssR binding box (TTTTTACA) which share 7 of 8 bases with the canonical sequence (TTTTCACA) [5]. Another putative CssR box (TTTTCAAA) is found between the start codon and the Shine–Dalgarno sequence but located on the other strand. The location and orientation of these putative CssR operator sites opens for speculations about CssRS being involved in regulation of *prsA* expression. A sensible notion is that CssRS calls for increased expression of the *prsA* foldase gene when poorly folded proteins are detected in the membrane/wall interface. In support of this theory we observed a twofold increase of native PrsA in the membranes of cells which over-express AmyL as compared to a *B. subtilis* reference strain. Overexpression of AmyL triggers the CssRS to two-component system to auto-phosphorylate CssR which then becomes able to activate transcription from the *htrA*- and *htrB*- promoters. While CssR is thought to act as a classical activator of the htrA/B promoters by binding to operators just upstream of their −35 boxes, the location of the putative CssR operators in the *prsA* P1 promoter region suggests that the regulator protein in the case of *prsA* regulation functions as a classical repressor. This could be by means of DNA looping facilitated by dimeric CssR or simply by monomeric CssR preventing access of the RNA polymerase to P1 when bound to its operators in the same promoter.

De-repression of a transcription site by CssR has already been reported in the case of the anti-adaptor protein YirB. The repressor YuxN forms a DNA loop by binding two boxes upstream and downstream of the *yirB* promoter. CssR P then binds a box that overlaps the upstream YuxN box, derepressing the *yirB* promoter [30]. Unless in *prsA*, in the case of *yirB*, the CssR P binding box is located upstream of the −35 site. In the gram-positive bacterium *Staphylococcus aureus*, PrsA is regulated by the VraRS two component system. Two TSS’s have also been identified in this case, one located 42 bp upstream of the ATG start codon, and another 139 bp upstream of ATG. The ATG proximal TSS has the VraR binding box on and upstream of the −35 site [12].

Overexpression of the native PrsA from *B. subtilis* increased the activity of AmyE, AmyS and AmyQ in the media in accordance with previous studies [19, 20], but had no significant effect on the other amylases tested in this work. While PrsA enhances productive secretion of enzymes like alpha amylases or subtilisin, it has no effect or even a detrimental one in the secretion of other proteins [20]. Thus, PrsA seems to have specificity for its substrates. As we have shown, the specificity of the PrsAs for its cognate amylase seems to be maintained in most cases even when expressed in a different host. In all cases, except in the case with the *G. stearothermophilus* pair, the cognate pairs gave the highest amylase yields or at least just as high as other non-cognate pairs. In the case of *G. stearothermophilus* amylase, though, co-expressing its cognate PrsA had no measurable effect on its secretion, but other PrsAs (*B. subtilis*, *B. licheniformis* and *B. amyloliquefaciens*) had. This observation illustrates the great potential of strain optimization prior to commercial exploration of microbial cell factories. *G. stearothermophilus* PrsA is phylogenetically the furthest away from the hosts PrsA. It is possible that although the *G. stearothermophilus* cognate amylase–PrsA pair would perform best in their original host, their interaction could be affected when moved to such a different host like *Bacillus subtilis*. The physicochemical properties around the membrane-cell wall interface could affect the nature of those interactions.

Overexpression of most heterologous PrsA proteins did not affect the growth profiles of the strains, except in the case of strains expressing PrsA^BN^. This could indicate that this particular PrsA might be very good at supporting amylase secretion but have a detrimental effect on other cell functions. As all strains were expressing both native- and heterologous *prsA* genes, two different homodimers and one heterodimer could potentially form. One of these combinations could increase folding efficiency for certain alpha-amylases, but reduce proper folding of other physiologically relevant proteins. The increase in cell lysis, a phenotype that is seen when PrsA is depleted or defective [19], might be a symptom of this.

In this work we measured the effect of heterologous PrsA over-expression on the secretion stress response of cells with forced alpha-amylase production. The intensity of the secretion stress response, defined as the level of activity of the CssRS regulon, was previously shown to be correlated with the level of AmyQ production [6]. Also, the introduction of the *prsA3* mutation, which reduces the level of PrsA more than tenfold, was shown to induce the secretion stress response in *B. subtilis*. This response was further increased if the *prsA3* mutation was combined with AmyQ production [26]. Thus, overexpression of amylase may impose stress to the cell and the intensity of this stress may be affected by the abundance, and perhaps also the nature of the co-expressed PrsA. In this study we show that heterologous expression of a PrsA in an alpha-amylase secreting strain could increase amylase secretion and at the same time decrease secretion stress. In an AmyL producing strain, the co-expression of *B. licheniformis* PrsA, B. sonorensis PrsA, and a mutated *B. licheniformis* PrsA decreased the secretion stress response while increasing amylase activity in the supernatant. A previous study showed that the secretion stress response, measured by a *htrB-lacZ* reporter gene fusion, decreased with the decrease in heterologous AmyQ secretion [31]. To our understanding, these two results are not contradictory: In the previous work, the decrease on AmyQ production was due to the lack of stability of the plasmid coding for *amyQ*, which would most probably result in a lower amount of AmyQ being produced, translocated though the Sec system, misfolded at the membrane-cell wall interface, leading to a decrease on CssS activation.

In our work, the chromosomal AmyL expression cassettes were the same in all strains, so the production and translocated amount of AmyL through the Sec system is expected to be the same. If the *prsA* co-expressed has a positive effect on the post-translocational folding of AmyL, more amylases would be rescued with less accumulation of misfolded proteins as a result. This would end in both an increase in the amount of secreted proteins and less stimulation of CssS. The recombinant PrsA^Bl-BN^, whose design was based on differences between the NC domains of the wild type foldases PrsA^Bl^ and PrsA^BN^, not only increased the measured activity of AmyL in the supernatant and decreased secretion stress, but also maintained a growth profile during the stationary phase similar to the parental *B. subtilis* 168 strain. This suggests that the cause of the detrimental interactions of PrsA^BN^ was not transferred to the recombinant PrsA^Bl-BN^. The substitution of only 6 amino acids around the NC domain affected significantly the efficiency of the folding and secretion of AmyL. The changed amino acids increased slightly the overall charge of this domain, which is very hydrophobic in the case of PrsA^Bl^. It is possible that a more charged NC domain affects the specific interaction between the unfolded amylase stretches and the PrsA positively. Since the primary physiological role of PrsA in the cell most likely is to fold PBPs and not amylases, it appears that there is room to improve this foldase for biotechnological purposes.

The positive effect that point mutations had on the effect of PrsA^Bl-BN^ compared to PrsA^Bl^ not only adds to the idea that the NC domain of PrsA could be important for substrate specificity, as suggested for the PrsA proteins of L. monocytogenes [14], but also opens a door for the design and improvement of PrsA.

## Experimental procedures

### Strains and growth conditions

*Bacillus subtilis* strains used in this study are listed in Additional file 1: Table S1. They are sporulation deficient derivatives of 168 (*trpC2*, *sigF*). AN2 is the parental strain which was used as a host for heterologous expression of proteins. Strains were cultivated at 37C in LB medium supplemented with chloramphenicol (6 $\mu$g/mL) or erythromycin (1 $\mu$g/mL) when appropriate. Competent cells and transformation of *B. subtilis* was obtained as described in Yasbin et al. [32]

### Construction of *B. subtilis* strains containing heterologous *prsA*- and/or *amy*- alleles

Gene Splicing by the Overlapping Extension (SOE) method [33] was used to generate linear recombinant DNA for transformation. Recombinant DNA was directed to a specific locus by addition of flanking regions containing sequences homologous to that locus. An antibiotic resistance marker gene was also included. Chromosomal integration was facilitated by homologous recombination and cells in where double cross over events occurred were selected for on LB agar plates containing the appropriate antibiotic. A PrsA expression cassette targeting the pel locus was assembled by use of the following DNA components: *pel* 5′ region (2.5 kb PCR product) + *ermC* (1.45 kb PCR product) + synthetic consensus promoter with SD sequence (*pconsSD*, 212 bp synthetic DNA) + *prsA* open reading frame with terminator (970 bp PCR product) + *pel* 3′ region (3.3 kb PCR product). Chromosomal DNA from AN2 was used as a template for PCR amplifications except for *ermC* where a Novozymes in-house plasmid served as a template. The resulting 8.73 kb SOE-PCR product thus targets the pel locus and contains the *prsA* expression cassette linked to an erythromycin resistance marker gene. This linear DNA was used directly for transformation of *B. subtilis* AN2 resulting in strain AQ34 which contains the PrsA(bs) expression cassette in pel locus. AQ34 chromosomal DNA then served as a master template for amplification of flanking regions used to direct *ermC* and heterologous *prsA* genes (orf exchange) to the pel locus of AN2. Templates for amplification of heterologous genes were either chromosomal DNA isolated from the indicated organism or synthetic DNA. Similarly, an expression cassette for the Ban-amylase (*amyQ*) targeting the *amyE* locus was assembled by use of the following DNA components: *amyE* 5′ region (2.8 kb PCR product) + synthetic consensus promoter with SD sequence (PconsSD, 172 bp synthetic DNA) + *amyQ* open reading frame with terminator (1.69 kp PCR product) + *cat* (1.2 kb PCR product) + *amyE 3*′ region (3.6 kb PCR product). Chromosomal DNA from AN2 was used as a template for PCR amplifications except for cat where a Novozymes in-house plasmid served as a template. The resulting 9522 bp SOE-PCR product thus targets the *amyE* locus and contains the *amyQ* expression cassette linked to a chloramphenicol resistance marker gene. This linear DNA was used directly for transformation of *B. subtilis* AN2 resulting in strain AQ1 which contains the AmyQ expression cassette in *amyE* locus. AQ1 chromosomal DNA then served as a master template for amplification of flanking regions used to direct cat and heterologous amylase genes (orf exchange) to the *amyE* locus of AN2. The native *amyE* becomes inactivated in this process. Templates for amplification of heterologous genes were either chromosomal DNA isolated from the indicated organism or synthetic DNA.

### Growth conditions, protein sample preparation, and mass spectrometry (MS)

*Bacillus subtilis* cells were grown aerobically at 37 °C in the presence of 20% glycerol in a synthetic minimal medium [34]. For the analysis of the membrane fraction, cells were harvested by centrifugation at early stationary phase. The cells were disrupted mechanically in a Precellys 24 homogenisator (PeqLab; 3 x 30 s at 6.5 $m s^-1$). Glass beads (0.1–0.11 mm diameter) and cell debris were removed by centrifugation (20,000x*g*, 10 min, 4 °C). Subsequently, the protein concentration of the samples was determined. Membrane proteins were enriched according to the protocol published in Eymann et al. [35]. All samples were analysed by the GeLC-MS workflow. After electrophoretic fractionation of each mixed sample by one-dimensional SDS-PAGE, gel lanes were sliced into 10 equidistant gel pieces followed by tryptic digestion as described by Eymann et al. [35]. For LC-MS/MS analyses of 1D gel samples, in-house self-packed columns were prepared and used with an EASY-nLC II system (Thermo). The peptides were loaded onto the column by the LC system with 10 $\mu$L of buffer A (0.1% (v/v) acetic acid) at a constant flow rate of 500 nL/min without trapping. The peptides were subsequently eluted using a nonlinear 100 min gradient from 1 to 99% buffer B (0.1% (v/v) acetic acid in acetonitrile) with a constant flow rate of 300 nL/min and injected online into the mass spectrometer. MS and MS/MS data were acquired with an LTQ Orbitrap XL (Thermo). After a survey scan at a resolution of 30 000 in the Orbitrap using lockmass correction, the five most abundant precursor ions were selected for fragmentation. Singly charged ions, as well as ions without detected charge states, were not selected for MS/MS analysis. Collision-induced dissociation (CID) fragmentation was performed for 30 ms with a normalized collision energy of 35, and the fragment ions were recorded in the linear ion trap.

Comparable protein amounts were calculated by the Hi3 method as described by Silva et al. [36]. For data processing and protein identification, raw data were imported into MaxQuant (1.6.3.3) where database search was carried against the respective B. subtilis strain with added contaminants from MaxQuant contaminant list with the following parameters: peptide tolerance: default, min fragment ions matches per peptide: 1, match between runs was enabled with the default settings, primary digest reagent: trypsin, missed cleavages: 2, variable modifications: oxidation M (+15.9949), acetylation N, K (+42.0106). Results were filtered on 1% FDR on spectrum, peptide and proteins level PSM. The peptide.txt file obtained from the MaxQuant (1.6.3.3, or above) search was loaded into R (v1.1.463); modified peptides were filtered out; peptide intensities of each biological replicate were separately normalized by division through the median, and the intensity sum of the three peptides of the corresponding protein with the highest normalized intensity were calculated, if at least three peptide intensities were reported in the corresponding biological replicate. A table containing these normalized intensities was exported to .xlsx format for each strain. A protein was only considered valid for quantification if values were existent in two out of 3 biological replicates [37]. In order to determine the relative amount of PrsA, we divided its intensity by the summed intensity of all quantified proteins. This value was then compared between all strains to see what are the changes between the expression of heterologous PrsAs.

### Construction of strains containing the *phtrA-lacZ* fusion

A LacZ expression cassette under the control of the *htrA* promoter and targeting the *xyl* locus was assembled by SOE PCR using the following DNA components (Fig. 6) : 5′*xyl* region (3.4kb PCR product) + spc (1.2 kb PCR product) + string A containing *phtrA* and the first $\frac{1}{2}$ of the *lacZ* gene (1.6kb PCR product) + string B containing the second $\frac{1}{2}$ of the *lacZ* gene (1.6 PCR product) + 3′*xyl* region (4.2 kb PCR product). Chromosomal DNA of AN2 was used as a template for PCR amplification of the 5′ and 3′ *xyl* regions and an in house plasmid was used as a template for the amplification of the spc resistance gene. The resulting PCR product was used direcly for transformation of strains AQG640, AQG492, AQG98, AQG77, AQG97, AQG174, AQG126 and AQG647 resulting in strains AQG735, AQG736, AQG737, AQG379, AQG741, AQG742, AQG745, AQG746 respectively, which express the *lacZ* gene under the *htrA* promoter.

### Microplate fermentation

The BioLector is a microfermentation system that monitors online common fermentation parameters such as biomass, pH, oxygen saturation and fluorescence. It contains a temperature and humidity controlled incubation chamber that carries a single microplate. The fermentation can be monitored continuously by an optical fiber that moves below the plate. In this work, a BioLector® (m2p-Labs, Baesweiler, Germany) was used for the measurement of scattered light and GFP fluorescence. Cultivations were performed in LB media, at a shaking frequency of 1000 rpm, 37 °C and 85% humidity in 48-well Flowerplates (M2p-labs), covered with a Sealing Foil with Reduced Evaporation (M2p-Labs). The fermentation was carried out in biological triplicates for 24 h, and the supernatant was harvested for subsequent amylase activity measurements.

### Amylase activity assay

Culture samples were measured for amylase activity in technical duplicates in 96 well plates. A calibration curve with increasing concentrations of BAN amylase (0–500 UCF/$\mu$L, Novozymes in-house product) was added to each 96 well plate. AmyL (Roche/Hitachi) Reagent 1 (66 mL) and Reagent 2 (16 mL) were mixed, and 180 $\mu$L of the mixture was added to the plate. The colorimetric reaction was measured in a Cytation5 plate reader at A405 nm, 23 °C for 6 min, measuring absorbance each minute. One unit of alpha amylase activity was defined as the amount of enzyme required to increase one unit of absorbance per minute under the assay conditions.

### β-Galactosidase assay

Cells were grown in 1.5 mL LB media in biological triplicates in Flowerplates at 37 °C and 1000rpm. After 24 h of growth, the OD600 was measured in technical duplicates, and the culture was transferred to 1.5 mL eppendorfs and centrifuged for 5 min at 15,000x*g*. The supernatant was discarded and the pellets were resuspended in 1 mL Z-buffer (10 mM DTT, 60 mM $Na_2HPO_4$, 40 mM $NaH_2PO_4$, 10 mM KCl, 1 mM $MgSO_4$, pH 7.0) and $10\mu \hbox {L}$ lysozyme (25 mg/mL) and incubated for 60 min at 37 °C and 700 rpm. After incubation, 2 technical replicates of $100\mu \hbox {L}$ each were transferred to new tubes, and 0.4 mL of ONPG was added to initiate the reaction. This was carried out at 30 °C and 700 rpm for 15 min when 1 mL $Na_2CO_3$ 1M was added to stop the reaction. After 15 min of stopping the reaction, the absorbance of the samples at 420 nm and 550 nm was measured. This experiment was done twice. One β-galactosidase activity units was defined as (Miller units: nmol $O.D.^{-1} min^{-1}$).

## Supplementary information

**Additional file 1.** Additional figures and table.

## Data Availability

All data generated or analysed during this study are included in this published article (and its additional files).
