# Supplementary material for: Identification and optimization of PrsA in Bacillus subtilis for improved yield of amylase
Source: Microb Cell Fact. 2019 Sep 17;18:158. doi: 10.1186/s12934-019-1203-0 (PMC6749698; doi:10.1186/s12934-019-1203-0)
Supplement: Supplementary file 1 — Additional file 1. Additional figures and table. [file 12934_2019_1203_MOESM1_ESM.docx]

## Additional Material

**Table S1. Strains used in this study**

***Strain name Relevant characteristic Source***

| *AN2* | *B. subtilis 168 ( ΔspoIIAc)* | Novozymes  internal |
| --- | --- | --- |
| *BS0675* | *B. amyloliquefaciens* | Novozymes  internal |
| *BS0861* | *G. stearothermophilus* | Novozymes  internal |
| *04JZM* | *B. licheniformis* | Novozymes  internal |
| *SJ36A* | *Bacillus sonorensis* | Novozymes  internal |
| *AQ1 AQ76 AQ77 AQ84 AQ587 AQ575 AQ34 AQ83 AQ126 AQ82 AQ131 AQ588 AQ567 AQ91 AQ108 AQ97 AQ99 AQ115 AQ610 AQ570 AQ92 AQ109 AQ98 AQ113 AQ100 AQ590 AQ572 AQ95 AQ694 AQ640 AQ645 AQ651*  *AQ592* | AN2 with integration of *amy::PconsSD-amyQ-cat* | This work |
|  | AN2 with integration of *amy::PconsSD-amyS-cat* | This work |
|  | AN2 with integration of *amy::PconsSD-amyL-cat* | This work |
|  | AN2 with integration of *amy::PconsSD-amyE-cat* | This work |
|  | AN2 with integration of *amy::PconsSD-NSP9.1 amy-cat* | This work |
|  | AN2 with integration of *amy::PconsSD-B. sonorensis amy-cat* | This work |
|  | AN2 with integration of *pel::erm-PconsSD-*BsubPrsA | This work |
|  | AQ34 with integration of *amy::PconsSD-amyE-cat* | This work |
|  | AQ34 with integration of *amy::PconsSD-amyL-cat* | This work |
|  | AQ34 with integration of *amy::PconsSD-amyS-cat* | This work |
|  | AQ34 with integration of *amy::PconsSD-amyQ-cat* | This work |
|  | AQ34 with integration of *amy::PconsSD-NSP9.1 amy-cat* | This work |
|  | AQ34 with integration of *amy::PconsSD-B. sonorensis amy-cat* | This work |
|  | AN2 with integration of *pel::erm-PconsSD- prsA^Bl^* | This work |
|  | AQ91 with integration of *amy::PconsSD-amyE-cat* | This work |
|  | AQ91 with integration of *amy::PconsSD-amyL-cat* | This work |
|  | AQ91 with integration of *amy::PconsSD-amyS-cat* | This work |
|  | AQ91 with integration of *amy::PconsSD-amyQ-cat* | This work |
|  | AQ91 with integration of *amy::PconsSD-NSP9.1 amy-cat* | This work |
|  | AQ91 with integration of *amy::PconsSD-B. sonorensis amy-cat* | This work |
|  | AN2 with integration of *pel::erm-PconsSD- prsA^Ba^* | This work |
|  | AQ92 with integration of *amy::PconsSD-amyE-cat* | This work |
|  | AQ92 with integration of *amy::PconsSD-amyL-cat* | This work |
|  | AQ92 with integration of *amy::PconsSD-amyS-cat* | This work |
|  | AQ92 with integration of *amy::PconsSD-amyQ-cat* | This work |
|  | AQ92 with integration of *amy::PconsSD-NSP9.1 amy-cat* | This work |
|  | AQ92 with integration of *amy::PconsSD-B. sonorensis amy-cat* | This work |
|  | AN2 with integration of *pel::erm-PconsSD- prsA^Gs^* | This work |
|  | AQ95 with integration of *amy::PconsSD-amyE-cat* | This work |
|  | AQ95 with integration of *amy::PconsSD-amyL-cat* | This work |
|  | AQ95 with integration of *amy::PconsSD-amyS-cat* | This work |
|  | AQ95 with integration of *amy::PconsSD-amyQ-cat* | This work |
|  | AQ95 with integration of *amy::PconsSD-NSP9.1 amy-cat* | This work |

*AQ565 AQ159 AQ178 AQ174 AQ199 AQ175 AQ606 AQ586*

| AQ95 with integration of *amy::PconsSD-B. sonorensis amy-cat* | This work |
| --- | --- |
| AN2 with integration of *pel::erm-PconsSD- prsA^BN^* | This work |
| AQ159 with integration of *amy::PconsSD-amyE-cat* | This work |
| AQ159 with integration of *amy::PconsSD-amyL-cat* | This work |
| AQ159 with integration of *amy::PconsSD-amyS-cat* | This work |
| AQ159 with integration of *amy::PconsSD-amyQ-cat* | This work |
| AQ159 with integration of *amy::PconsSD-NSP9.1 amy-cat* | This work |
| AQ159 with integration of *amy::PconsSD-B. sonorensis amy-*  *cat* | This work |
| AN2 with integration of *pel::erm-PconsSD- prsA^Bson^* | This work |
| AQ162 with integration of *amy::PconsSD-amyE-cat* | This work |
| AQ162 with integration of *amy::PconsSD-amyL-cat* | This work |
| AQ162 with integration of *amy::PconsSD-amyS-cat* | This work |
| AQ162 with integration of *amy::PconsSD-amyQ-cat* | This work |
| AQ162 with integration of *amy::PconsSD-NSP9.1 amy-cat* | This work |
| AQ162 with integration of *amy::PconsSD-B. sonorensis amy-*  *cat* | This work |
| AN2 with integration of *pel::erm-PconsSD-prsA^Bl-BN^* | This work |
| AQ462 with integration of *amy::PconsSD-amyL-cat* | This work |
| AQ640 with integration of *xyl::PhtrA-lacZ-spec* | This work |
| AQ492 with integration of *xyl::PhtrA-lacZ-spec* | This work |
| AQ98 with integration of *xyl::PhtrA-lacZ-spec* | This work |
| AQ77 with integration of *xyl::PhtrA-lacZ-spec* | This work |
| AQ97 with integration of *xyl::PhtrA-lacZ-spec* | This work |
| AQ174 with integration of *xyl::PhtrA-lacZ-spec* | This work |
| AQ126 with integration of *xyl::PhtrA-lacZ-spec* | This work |
| AQ657 with integration of *xyl::PhtrA-lacZ-spec* | This work |

*AQ162 AQ662 AQ657 AQ668 AQ672 AQ667 AQ661*

*AQ462 AQ492 AQ735 AQ736 AQ737 AQ739 AQ741 AQ742 AQ745 AQ746*


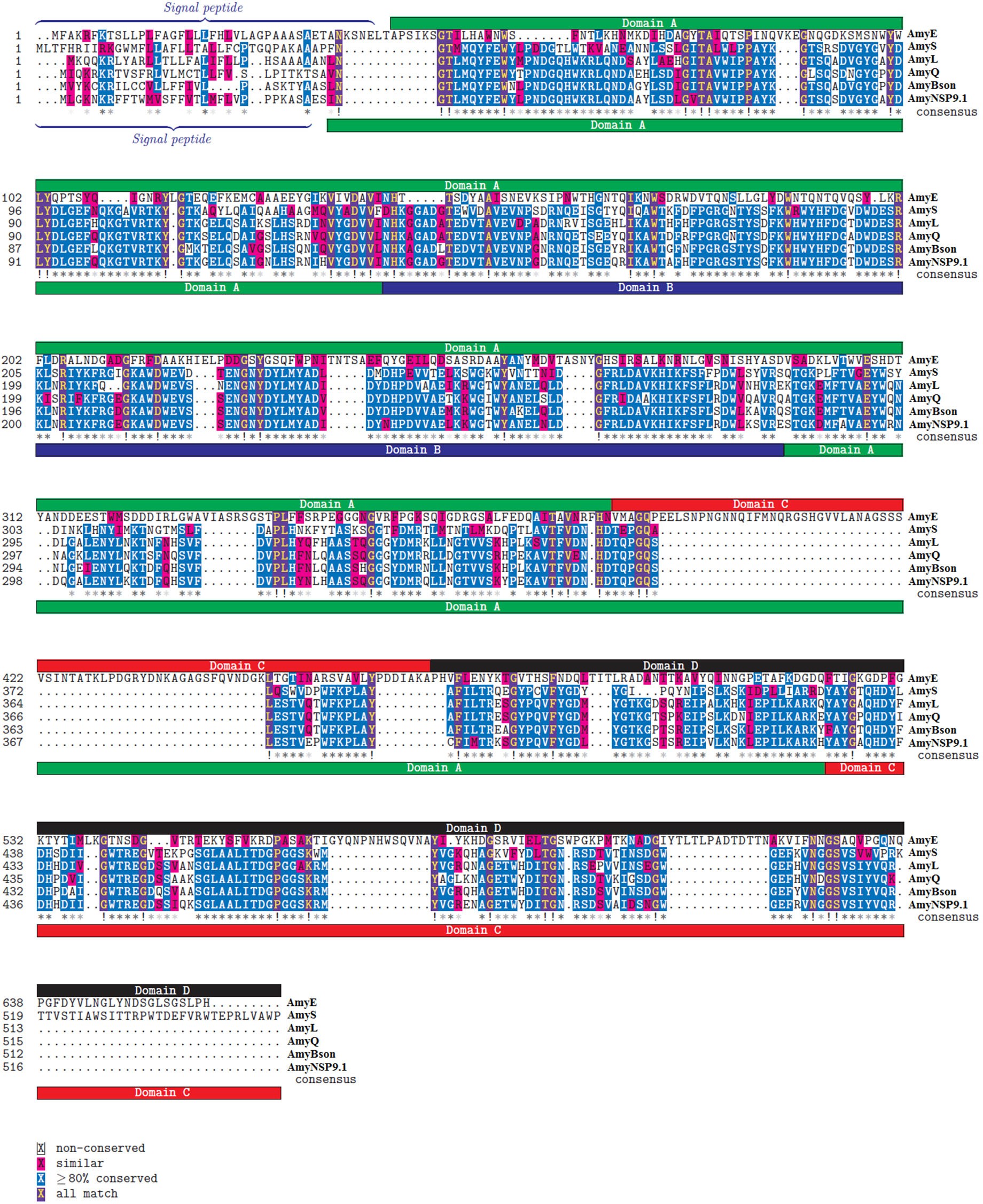


**Figure S1. Protein sequence alignment of the alpha-amylases used in this study.** The domains which correspond to the alpha-amylases belonging to the GH13_5 subfamily are presented on the bottom of the sequences and the domains which correspond to the B. subtilis amylase AmyE, which belongs to the GH13_28 subfamily, are presented on the top of the sequences.


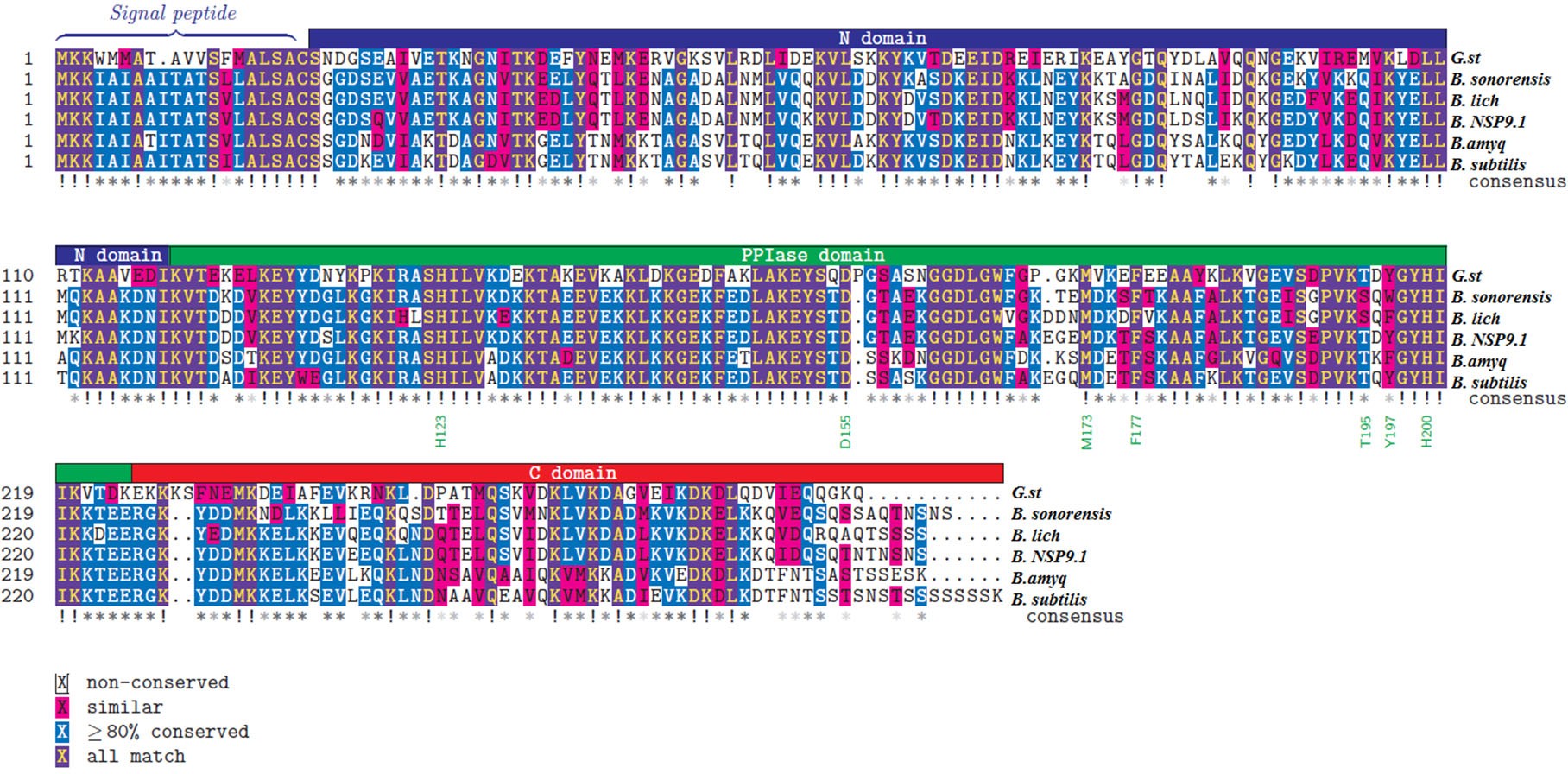


**Figure S2. Protein sequence alignment of the PrsA proteins used in this study.** The domains are


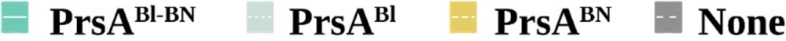


presented on the top of the sequences and residues that belong to the active site of the peptidyl prolyl cis/trans isomerase domain (PPIase) are shown in green on the bottom.


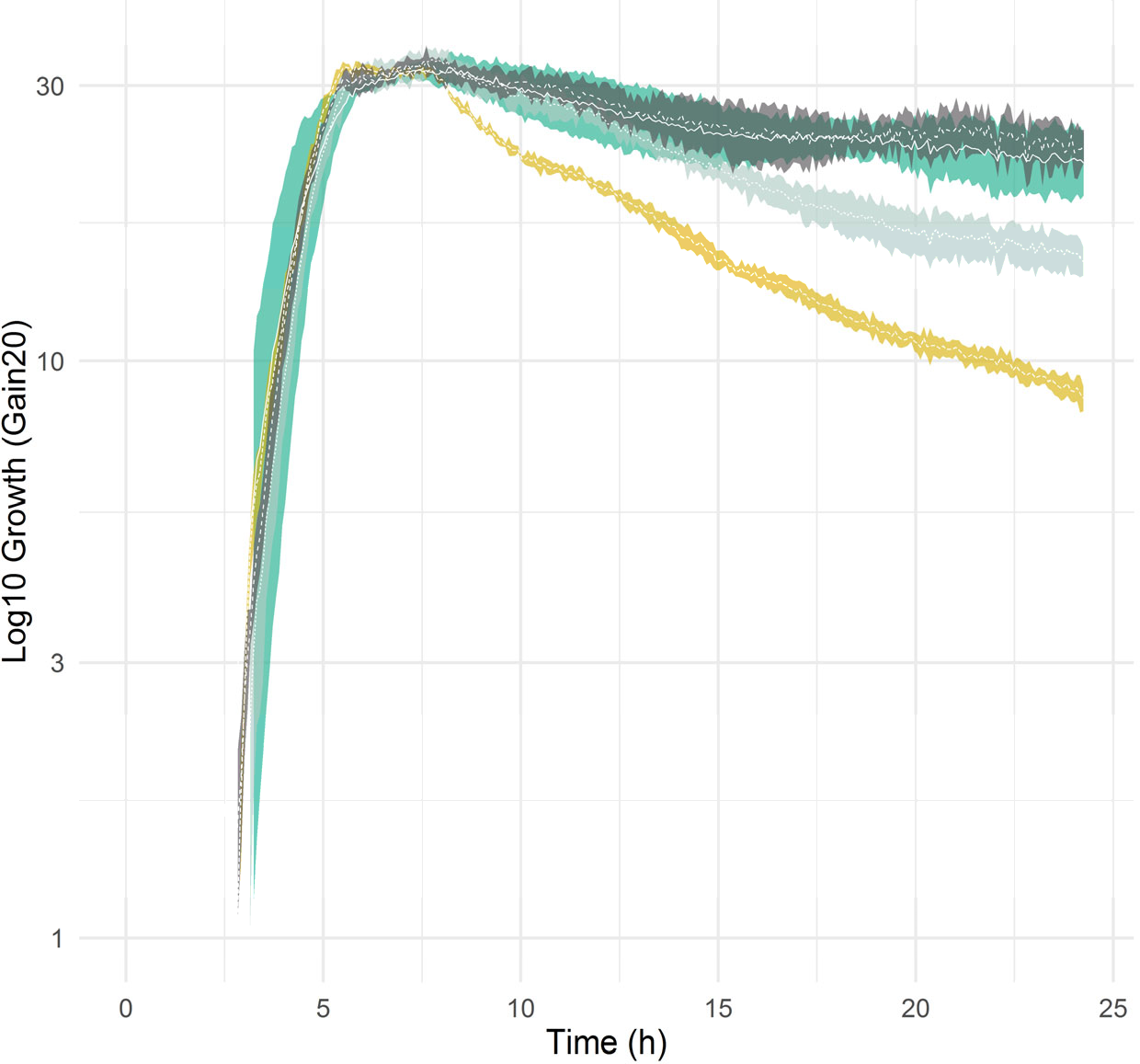


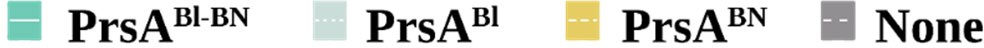


**Figure S3. Growth profiles.** Cultivations were performed in a Biolector microreactor measuring culture density on-line as light scattering. Cultures were performed in biological triplicates and the average optical density of the three cultures is presented by a white line. The coloured area around the lines shows the extent of the standard deviation between the three replicates. All strains cultivated are expressing *amyL* and the additional PrsA showed below the figure.
